# Hyperinflammatory Immune Response and COVID-19: A Double Edged Sword

**DOI:** 10.3389/fimmu.2021.742941

**Published:** 2021-09-30

**Authors:** Li Yin Tan, Thamil Vaani Komarasamy, Vinod RMT Balasubramaniam

**Affiliations:** ^1^ Infection and Immunity Research Strength, Jeffrey Cheah School of Medicine and Health Sciences, Monash University Malaysia, Bandar Sunway, Malaysia; ^2^ Greenslopes Private Hospital, Greenslopes, QLD, Australia

**Keywords:** COVID-19, SARS-CoV-2, immunopathogenesis, hyperinflammation, cytokine storm, innate immune response, adaptive immune response

## Abstract

The coronavirus disease-19 (COVID-19) elicited by the severe acute respiratory syndrome coronavirus 2 (SARS-CoV-2) has caused devastating health, economic and social impact worldwide. Its clinical spectrum ranges from asymptomatic to respiratory failure and multi-organ failure or death. The pathogenesis of SARS-CoV-2 infection is attributed to a complex interplay between virus and host immune response. It involves activation of multiple inflammatory pathways leading to hyperinflammation and cytokine storm, resulting in tissue damage, acute respiratory distress syndrome (ARDS) and multi-organ failure. Accumulating evidence has raised concern over the long-term health effects of COVID-19. Importantly, the neuroinvasive potential of SARS-CoV-2 may have devastating consequences in the brain. This review provides a conceptual framework on how the virus tricks the host immune system to induce infection and cause severe disease. We also explore the key differences between mild and severe COVID-19 and its short- and long-term effects, particularly on the human brain.

## Introduction

The severe acute respiratory syndrome coronavirus 2 (SARS-CoV-2) is a novel coronavirus first discovered in Wuhan on the 31^st^ of December 2019. Known as the third highly infective CoV, it has a high transmissibility capacity. It has a basic reproduction number (R0) of 2.2 and a 2% mortality rate (1). Due to the rapid spread of this virus across the globe, the World Health Organisation (WHO) declared Coronavirus disease 2019 (COVID-19) as a global pandemic on the 11^th^ of March 2020 (2). As of the 8^th^ of September 2021, there are currently 221,648,869 confirmed cases of COVID-19 and 4,582,338 deaths reported (3).

SARS-CoV-2 is an enveloped, positive-sense, single-stranded RNA virus belonging to the Betacoronaviridae family (4). It has a spherical shape of 60-140nm in diameter with characteristic club-shaped spikes on the outer surface of the virion (1, 4). This solar corona appearance led to the name coronavirus (1, 4). The SARS-CoV-2 genome sequence comprises the following: non-coding 5’-untranslated region (UTR)-replicase gene (ORF1ab), structural proteins (spike, envelope, membrane and nucleocapsid), accessory proteins (ORF6, 7ab, 8 and 9b) and non-coding 3’-UTR (5). It consists of 14 open reading frames (ORFs) encoding a total of 27 proteins (5). Each protein has its own unique role in viral infectivity. For instance, the spike (S) glycoprotein consists of the S1 subunit, which aids in viral recognition and attachment, and the S2 subunit that facilitates membrane fusion between virus and host (6, 7). On the other hand, E protein, the smallest structural protein, facilitates transportation and recruitment of the virus; M protein assists morphogenesis; N protein involves in RNA production (6).

Since SARS-CoV-2 has an incubation period of around 5-6 days and up to 14 days, the virus can easily be transmitted from one person to another during this time. Although the majority of the infected population may exhibit mild to moderate illness, others in particular the elderly, may experience complications from the disease resulting in death (8). COVID-19 can be categorised into mild, moderate, severe and critical (**Table 1**).

**Table 1:** Classification of COVID-19.

Classification	Mild	Moderate	Severe	Critical	Ref.
Signs & symptoms	Mild symptoms	Fever	Respiratory distress ≥30 breaths/min	Respiratory failure and requiring mechanical ventilation	(9)
		Respiratory symptoms	Resting SpO2 ≤ 93%	Shock	
			Arterial partial pressure of oxygen (PaO2)/fraction of inspired oxygen (FiO2)≤300mmHg (l mmHg = 0.133 kPa)	With another organ failure necessitating ICU care	
Imaging	No signs of pneumonia	Signs of pneumonia present	Obvious lesion progression within 24–48h >50%	Diffuse pulmonary involvement (“white lung”)	(9, 10)
Pathogenesis	Increased viral replication	Viral replication continues	Predominant inflammatory response	Inflammatory response	(11, 12)
		Inflammatory response	Reduced viral replication		
Therapeutic Intervention	Antiviral drugs	Antiviral drugs	Anti-inflammatory drugs	Anti-inflammatory drugs	(11, 12)
	Antibody therapy	Antibody therapy	Antiviral drugs		

As COVID-19 is a novel disease, the long-term clinical implications of COVID-19 remain to be elucidated. The increasing reports on post-acute sequelae have raised concern over the possible burden of COVID-19 chronicity, especially on the neurological system (13, 14). In this study, we aimed to review the current understanding of COVID-19 pathogenesis, the difference between severe and non-severe stage of disease, as well as its short- and long-term effects, particularly on the human brain.

## Immunopathogenesis of SARS‐CoV‐2

### Innate Immunity

**Figure 1:**
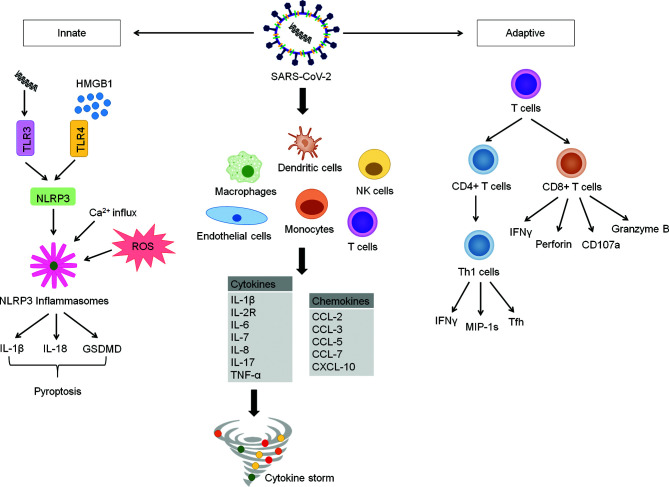
COVID-19 Inflammatory Signaling Pathway. COVID-19 is characterized by delayed intracellular innate immune responses associated with type I and type III IFNs. This allows for rapid virus replication and subsequent PRR-induced abnormal inflammatory response. SARS-CoV-2 invade and activate of various immune cells such as endothelial cells, macrophages, monocytes, dendritic cells, natural killer cells and T-cells. These abnormally activated immune cells stimulate multiple inflammatory pathways producing large amounts of cytokines and chemokines, which results in hyperinflammation and cytokine storm. The formation of cytokine storm reduces further spread of virus in the body but also causes tissues damage, causing acute respiratory distress syndrome (ARDS) and multi-organ failure. Upon entering cells, SARS-COV-2 is recognized by pattern-recognition receptor (toll-like receptor 3), resulting in activation of NLRP3. Simultaneously, the viral invasion causes protein aggregation, calcium influx and ROS formation. These events lead to the initiation of NLRP3 inflammasome. Activated NLRP3 inflammasome stimulates activation of caspase-1, which cleaves Gasdermin D (GSDMD), resulting in pyroptosis of cells. Activated caspase-1 also promotes the secretion of IL-1β, IL-18, which play an important role in pyroptosis procession. HMGB1, a prototypical DAMP activates inflammasome and play an essential role in the inflammatory response in COVID-19 patients. The adaptive immunity kicks off soon after the trigger of innate immune response to control virus infection through antibody-producing B cells, CD4+ T cells and CD8+ T cells. CD4+ T cells are important for virus clearance, while CD8+ T cells provide protection against the virus through multiple cytokines. However, CD4+ and CD8+ T cells are found to be significantly reduced in patients with severe COVID-19.

SARS-CoV-2 entry into the host cell is initiated by binding of the receptor-binding domain (RBD) in the S1 subunit to host cell surface receptor, angiotensin-converting enzyme 2 (ACE2). The viral attachment step is followed by the priming of S protein by a cellular serine protease, TM protease serine 2 (TMPRSS2), which facilitates fusion of viral and host membranes through the S2 subunit (15). ACE2 and TMPRSS2 were found to be highly expressed in lung epithelial cells (15–17).Upon entering the target cell, SARS-CoV-2 RNA is recognised by a pattern-recognition receptor (PRR), toll-like receptor 3 (TLR3), and this activates the transcription of NLR family pyrin domain containing 3 (NLRP3) gene (18). At the same time, in response to the viral invasion, protein aggregation, calcium flux from the cytoplasm, and reactive oxidative species (ROS) formation occur (18). Together, these responses culminate in the initiation of the NLRP3 inflammasome, inducing downstream signaling inflammatory and coagulation cascades (18). Furthermore, these changes involve the secretion of interleukin-18 (IL-18), interleukin-1β (IL-1β) and gasdermin D (GSDMD) extracellularly (**Figure 1**) (18). These events subsequently result in pyroptosis of cells which is also evident by the increase in the plasma lactate dehydrogenase in COVID-19 patients (18, 19).

The damage-associated molecular pattern (DAMP) High-Mobility Group Box 1 (HMGB1) has been found to assist SARS-CoV-2 transfer into the cytoplasm in the post-entry step and contributes to viral replication (20). It is also suggested that a high concentration of HMGB1 passively secreted from necrotic or activated cells further promotes inflammation (**Figure 1**) (21). In the context of COVID-19-induced neuroinflammation, HMGB1 acts on the TLR4 receptors expressed on neurons, microglial cells, and astrocytes, effectually generating cytokine release (21).

During SARS-CoV-2 invasion, early activation of type I IFN is vital as it provides an immediate suppression of viral replication (22). Notably, SARS-CoV-2 can selectively counteract the host cell’s interferon signaling pathway, thereby evading the immune system (18). It possesses various structural and non-structural proteins that can subvert the type I Interferon (IFN) responses (23). Although the exact molecular interaction remains to be determined, inhibition of the type 1 IFN is postulated to occur at various stages, including suppressing the PRR recognition of SARS-CoV-2 RNA, downstream PRR or interferon signaling, as well as preventing translation *via* degradation of host cell mRNA (23). The resultant suppressed or delayed type I IFN cascade enhances viral replication and causes inflammatory cytokine storm (18).

In addition, viral proteins and inflammatory cytokines induce neutrophil activation, leading to ROS secretion and the formation of neutrophil extracellular traps (NETs) (24, 25). HMGB1, which is triggered by ROS, may also play a role in NET activation (26). Increased concentration of NETs has been observed in plasma, tracheal aspirate, and lung specimens of autopsies from COVID-19 patients (27). NETs further promote and sustain the local inflammation. A high concentration of NETs positively correlates with sepsis severity and organ dysfunction, and they have been shown to contribute to immunothrombosis in the course of inflammatory response (27–30). These early events that involve interaction between SARS-CoV-2 with host cells including, innate immune cells play an important role in inducing endothelial damage, acute lung injury, disruption of lung structure associated with pulmonary edema and pneumonia, multi-organ damage and death in COVID-19 disease.

### Adaptive Immunity

Soon after the trigger of the innate immune response, antibody-producing B cells, CD4+ T cells and CD8+ T cells of the adaptive immunity are primed to control pathogenic infection. While innate immunity is intrinsically involved in COVID-19 immunopathogenesis, there is limited evidence supporting the pathogenic phenomenon of adaptive immunity. In contrast to massive innate cytokines or chemokines associated with immunopathology, elevated T cells are therapeutic and do not worsen the disease. Seroconversion is shown to occur in more than 90% of COVID-19 patients a few weeks post-infection (31). Compared to CD8+ T cells, CD4+ T cells present a greater antiviral effect towards SARS-CoV-2 infection and better control of disease severity (32, 33). The primary targets of CD4+ T cells include the highly expressed spike, M and nucleocapsid antigen, with significant specificity for nsp3, nsp4 and ORF8 (32). Meanwhile, CD8+ T cells showed a slightly different immunoreactivity, with spike protein, nucleocapsid, M, nsp6, ORF8 and ORF3a being the target antigens (32).

Detected as early as 2 to 4 days after onset of COVID-19 symptoms (33), CD4+ T cells play an important role in assisting the development of CD8+ cells and B cells. They can differentiate into T helper 1 (Th1) cells, which generate antiviral IFNγ and other cytokines, as well as T follicular helper (Tfh) cells that facilitate the majority of the neutralizing antibody production (**Figure 1**) (34). Moreover, they express high levels of chemokine gene CCL3/4/5 (MIP-1s) and XCL1, thereby recruiting other relevant immune cells to the site of viral antigen (35). Likewise, CD8+ T cells can develop quickly post-infection as early as one day after symptom onset (36). CD8+ T cells produce various potent cytotoxic molecules capable of clearing infected cells, such as IFNγ, perforin, CD107a and granzyme B (**Figure 1**) (37). Neutralizing antibodies produced by naïve B cells act on the spike and nucleocapsid of SARS-CoV-2 (38). Reports have demonstrated the concurrent development of IgG, IgA and IgM against the spike antigen. The RBD of spike presents as the target of most of the neutralising antibodies (39–41). Although SARS-CoV-2 neutralising antibody levels have not been correlated with reduced disease severity (42), circulating Tfh cells, the precursors to the neutralising antibody response, are capable of doing so (33). This could be due to a slower adaptive immune response where the host is already infected severely.

### Cytokine Release Syndrome

Cytokine release syndrome, also known as cytokine storm, correlates with COVID-19 severity and has been recognised as a major cause of mortality among COVID-19 patients (43). It is defined as a life-threatening condition involving the excessive cytokine and chemokines produced by the dysregulation of the immune response (43). Considering that these inflammatory mediators are interdependent and can be both protective and pathologic, distinguishing them can be challenging. In the serum of COVID-19 patients with cytokine storm, various raised cytokine levels are reported. This includes IL-1β, interleukin-6 (IL-6), tumor necrosis factor (TNF), macrophage inflammatory protein (MIP) 1α and 1β, interferon-γ, inducible protein 10 (IP-10), and VEGF (19, 44).

The underlying mechanism is complex. What is known is that the dysfunction of IL-6/Janus kinase/signal transducer and activator of transcription (IL-6/JAK/STAT) signaling pathway, interferon (IFN) cell signaling cascade, TNF-α-nuclear factor-kappa (TNFα-NF-κB) pathway, toll-like receptor (TLR) pathway, antibody-mediated pathway, Bruton tyrosine kinase (BTK) pathway, renin-angiotensin system (RAS) pathway and especially the Janus kinase signal transducer and activator transcription (JAK/STAT) pathway, are all integral to the progression of cytokine storm (45). In general, SARS-CoV-2 invades and activates a myriad of immune cells, such as endothelial cells, macrophages, monocytes, dendritic cells, natural killer cells and T-cells (45). In these cells, the viral particles stimulate multiple inflammatory pathways, producing low levels of antiviral interferons alongside high levels of cytokines (IL-1β, IL-2R, IL-6, IL-7, IL-8, IL-17 and TNF-α) and chemokines (CCL-2, CCL-3, CCL-5, CCL-7, CXCL-10), resulting in hyperinflammation and cytokine storm (**Figure 1**) (45). While the initial release of cytokines is therapeutic and aid in eliminating the virus, the uncontrolled cytokine secretion would be detrimental as they begin to target host cells (45, 46). Among the cytokines, IL-1β, IL-2R, IL-6, and TNF-α play a pivotal role in cytokine storm while IL-1β, IL-6, and TNF-α correlate well with the severity of disease (45, 47). On the other hand, the weakened T-cell immune effect further hamper viral elimination, allowing a vicious cycle of cytokine-induced hypercytokinaemia (48). A more detailed representation of the inflammatory signaling pathway involved in COVID-19 is shown in **Figure 1**.

## Gender Difference in Severity and Mortality of COVID-19

Increasing evidence support that compared to women, men exhibit more severe morbidity and higher mortality upon COVID-19 infection (49–51). In addition, men with COVID-19 had higher rates of intensive care unit (ICU) admissions and were more likely to require mechanical ventilation (52, 53). The sex-based disparities in COVID-19 severity and mortality are most likely attributed to pre-exiting comorbidities, social and lifestyle factors, host immune response, genetic constitutions, hormone milieu and sex organs (53–56).

Women have a stronger antigenic response to infections and vaccinations, as well as a higher risk of developing autoimmune disease compared to men (57, 58). Hence, it is possible that women may possess enhanced inflammatory regulation and antiviral defense. A study found that female COVID-19 patients had more robust T cell activation than male patients. A lower proportion of activated T cells in males was associated with disease progression (56). In addition, infected males have exhibited higher levels of pro-inflammatory cytokines and chemokines such as IL-8, IL-18 and CCL5, which can contribute to increased cytokine storm and a higher risk of poor disease outcomes (56).

Other than that, different hormonal milieus between men and women may potentially have a role in viral infections. Men exhibit lower innate immune response due to lack of early estrogen activated TLR7 antiviral activity as well as low stress endurance causing higher cellular necrosis and therefore greater HMGB1 release. Other speculations include immunosuppressive effects by testosterone and high viral expressions in testes delaying viral clearance from the body (55). In contrast, oestradiol in females offers protection against COVID-19 *via* enhancement of the body’s innate and adaptive immune responses through neutrophils, cytokines and antibody production (51).

Although global data have shown higher mortality rates among males than women, India demonstrated the opposite. The data from India may possibly be hampered by the differences in socioeconomic status and access to healthcare between both genders (59). Therefore, further analyses on sex-aggregated data are required to generate reliable findings of gender influence on COVID-19 severity.

## Long-Term Sequelae of Mild-Moderate vs. Severe COVID-19

Long-COVID or long-hauler is a given term for patients whose symptoms persisted or developed following COVID-19 infection resolution (14). The majority of the symptoms in the post-convalescent period were comparable to the acute phase of COVID-19 (**Table 2**) (14). Many existing literatures have focused on follow-up in recovered patients 1 to 3 months post-infection (67–69). Symptoms reported in descending frequency include fatigues, dyspnea, joint pain, chest pain, cough, anosmia, sicca syndrome, rhinitis, red eyes, dysgeusia, headache, sputum secretion, appetite loss, sore throat, vertigo, myalgia and diarrhea (67). Hair loss, attention disorder, memory loss and sleep disorder were also reported (68).

**Table 2:** Comparison between non-severe and severe COVID-19.

Stage of Initial Disease	Mild-Moderate	Severe	Ref.
Symptoms	Fever, cough, fatigue, myalgia, dyspnea	Fever, cough, fatigue, myalgia, dyspnea, acute respiratory distress syndrome (ARDS) and multiple organ failure	(48, 60)
Cytokine levels	Elevated cytokines	Highly elevated cytokiens (cytokine storm)	(48, 60)
	↑IL‐6, IL‐10 and TNF‐α	↑↑IL-2, IL‐6, IL-7, IL-8, IL‐10, IL-1β, IL-2R, TNF‐α and MCP‐1	
T cell lymphopenia	↓Lymphocytes (CD4+T and CD8+T cells)	↓↓Lymphocytes (CD4+T cells and CD8+T cells)	(48, 60, 61)
		↓IFNγ expressing CD4+T cells	
Post-COVID consequences at risk	Fatigue, muscle weakness, sleep disturbance, anxiety, depression, persistent renal impairment, anosmia & ageusia, shortness of breath, brain fog	Fatigue, muscle weakness, sleep disturbance, anxiety, depression, anosmia & ageusia, shortness of breath, brain fog.	(62–65)
		Pulmonary diffusion impairment & abnormal radiological imaging (ground glass opacity & irregular lines)	
Risk of reinfection	Modest-Significant decline in neutralising antibodies titres & seropositivity	Significant decline in neutralising antibodies titres & seropositivity	(63, 66)

In the longest follow-up study to date (up to 9 months), persistent symptoms including fatigue (13.6%), anosmia or ageusia (13.6%), and brain fog (2.3%) were reported (62). About a third of patients also reported a decline in health-related quality of life due to COVID-19 compared to the baseline level (62). A 6-months study on 1733 COVID-19 patients yields similar results, with 76% of the patients had at least one symptom, with the commonest being fatigue, muscle weakness or sleep disturbances (63). The severity of disease and female gender were identified as risk factors for post-COVID-19 consequences (63). Another 3-months study on COVID-19 patients revealed that females were more susceptible to fatigue, post-activity polypnea and hair loss compared to males (64).

Interestingly, a medium-term (2.5 months or 75 days) follow-up on patients found a lack of association between initial disease severity and post-COVID morbidities such as fatigue, pulmonary abnormalities or clinical fibrosis (65). Instead, the susceptibility of post-COVID morbidity is dependent on the length of hospital stay (65). Nevertheless, further investigations are required to delineate both variables as initial disease severity may be closely linked to the length of hospitalization (65).

## Neuro-COVID-19

### Route of Entry Into the Brain

COVID-19 has been widely known to infect the human respiratory system. However, several anecdotal reports on neurological manifestations such as headache, dizziness, altered mental state, acute cerebrovascular disease, and meningoencephalitis have emerged (70–73). Neurological involvements were more susceptible among those with severe infections and were the only initial presenting symptoms in some (70). Given the wide range of symptoms associated with COVID-19 as time unfolds, the effects of COVID-19 can be expected to occur through multiple neuro-invasive pathways. Emerging studies have described the underlying pathways *via* the direct (neurotropism of SARS-CoV-2) and indirect route (due to inflammation, hypoxia, thrombosis and imbalance in blood pressure). Additionally, ACE2 receptors, the principal viral entry receptor for SARS-CoV-2, are found in both neurons and non-neuronal cells (astrocytes, endothelial cells and oligodendrocytes) of the central nervous system (CNS) (17). Multiple CNS regions with high expression of ACE2 receptors include the amygdala, cerebral cortex and brainstem (74).

Earlier symptoms, including impaired sense of smell detected in a significant number of COVID patients strongly indicate the viral potential for olfactory transmucosal invasion into the CNS (75–78). An animal study whereby intranasal administration of SARS-CoV-2 virus into K18-hACE2 mice demonstrated an infective progression with high viral RNA levels in nasal turbinates, followed by olfactory bulbs and eyes (79). The spread of infection into the brain may be explained by the high viral replicative rate in these tissues and subsequent direct infection of the adjacent neuron axons (79). Peak viral levels in the brain were about 1000 times higher, whereas the production of pro-inflammatory cytokine and chemokine mRNAs in the brain was about 10-50 times higher compared to the lungs (79). Brain sections of mice showed perivascular hemorrhage, increased leukocyte infiltration and neuronal cell degeneration (79). These results correlated with the clinical severity and mortality of the infected mice (79). Evidence from a post-mortem autopsy on 33 COVID19-infected individuals showed intact SARS-CoV-2 particles and its RNA in olfactory mucosa and other neuroanatomical regions receiving the axonal projections, supporting the notion of neuroinvasion *via* neural-mucosal barrier (80).

Cerebellum, the CNS region independent from olfactory mucosa, has also shown infection with SARS-CoV-2, suggesting other possible ports of entry into the CNS apart from axonal transport (80). A recent study demonstrated that the S1 subunit of SARS-CoV-2 spike protein is able to penetrate the blood-brain barrier (BBB) in mice when administered intravenously and with a greater rate and level of uptake compared to the nasal route (81). The mechanism involved absorptive transcytosis *via* attachment to cellular surface glycoproteins comprising N-acetylglucosamine or sialic acid and equal distribution of S1 is found across the whole brain. This BBB is further impaired by the hyperinflammatory effect of COVID-19 (81).

Consistent with the notion that COVID-19 induces a hyperimmune response, this cytokine overproduction may underlie the potential mechanism into the CNS, possibly *via* the crossing of BBB. Previous studies corroborated this indirect mechanism, where high autoantibodies levels are detected in the cerebrospinal fluid of COVID-19 patients with neurological manifestations (82–86). It appears, therefore, that an indirect immune-mediated mechanism may represent a preferential gate to the brain. However, further research is warranted to clarify the various unknown autoantigens in the CNS that are targeted. Additionally, the hypoxic state in severe COVID-19 patients, especially those with acute respiratory distress syndrome, may cause oxygen deficiency and anaerobic metabolism to occur in the brain (87). This acid accumulation, in turn, leads to neuronal ischemia, interstitial edema, cerebral obstruction and vasodilatation, causing acute cerebrovascular disease and CNS injury (87). A report of intracerebral hemorrhage in COVID-19 patients points to the potential mechanism of endothelial impairment (88). Owing to the fact that ACE2 receptors are present in cerebrovascular endothelial cells and modulate blood pressure in the renin-angiotensin-aldosterone system, SARS-CoV-2 may target these receptors, disrupting the blood pressure leading to hypertension or hypotension (87–89).

Patients with COVID-19 also share similar coagulation markers as other forms of coagulopathy, such as disseminated intravascular coagulation and sepsis-induced coagulopathy, with elevated D-dimer, fibrinogen, prothrombin time, von Willebrand factor, as well as reduced thrombocytes (90–93). These findings shed light on the prothrombotic nature of COVID-19, attributed to both hemostasis and immunothrombosis (90). COVID-19-induced endothelial dysfunction exposes the subendothelial collagen and tissue factor, which potently activates the coagulation pathway and platelet aggregation, resulting in thrombus formation (90). Simultaneously, the thrombin and factor Xa can activate innate immunity, while fibrinogen and fibrin can activate neutrophils (90). Following that, the cytokine secretion may then promote platelet aggregation together with the help of NETs. The membrane attack complexes may also initiate microthrombi and von Willebrand factor formation *via* host cell death (90). Eventually, all these immune responses would enhance the tissue factor pathway and complement hemostasis (90).

### Effects on the Brain

With the discovery of the neuroinvasive potential of COVID-19 and the devastating consequences in the brain, there are concerns over the possibility of long-term neurological and neuropsychiatric sequelae. A recent systematic review and meta-analysis identified several long-term neuropsychiatric effects such as headache, attention disorder, anosmia, memory loss, brain fog, neuropathy, anxiety, depression, insomnia, dementia, dizziness, stroke, dysphoria, obsessive-compulsive disorder, post-traumatic stress disorder and paranoia (14).

Histopathological changes of the human brain, including cerebral micro thrombosis and acute cerebral infarcts, mediated by neuroinflammation and immunoreactivity, have been reported in post-mortem autopsies (80). Using SARS-CoV-2-infected human brain organoid, SARS-CoV-2 is seen to exploit host cell metabolic machinery for its replication, promoting a hypermetabolic and hypoxic environment, resulting in neuronal death in infected and adjacent cells (94). The study further demonstrated significant disturbance of brain vasculature in mice models, which may explain the association between hypoxia and micro-ischemic damage found in human organoid and post-mortem brain autopsies (94).

Type I Interferon (IFN) are the common cytokines generated in response to viral infection (95, 96). In view of the mounting evidence on COVID-19-related cytokine storm, a similar phenomenon is postulated in COVID-19 infection. According to a study by Roy et al. the IFNs are observed to directly activate CNS microglia and promote the complement cascade (95, 96). The nucleic acid-containing amyloid fibrils enveloped by the microglia is found to upregulate gene expression involved in IFN interaction (95, 96). Moreover, the amyloid fibres may capture SARS-CoV-2 viral particles and contribute synergistically to the augmentation of IFN effect (95, 96). This resulted in synaptic loss, commonly seen in Alzheimer’s Disease (95, 96).

## Therapeutic Modulation of Primary Cytokines in COVD-19

Based on the strong association between elevated levels of the cytokines and severity of COVID-19, modulation of inflammatory cytokines provides therapy strategies to mitigate severe disease. Cytokine-targeted therapies have become the preferable option as they have fewer potential adverse effects compared to those broad, non-specific therapies that target multiple cytokine pathways, for instance intravenous immunoglobulin, corticosteroid, cDK7 inhibitor and certain traditional Chinese medicines. Many cytokines, including IL-1, IL-6, IL-17 and TNF have been investigated as pathological targets for treatment.

Multiple studies have found that IL-1 inhibitors, Anakinra and Canakinumab are effective in improving patients’ clinical conditions as well as their biochemical profiles. Proposed mechanisms of IL-1 blockade involved prevention of immune cells migration to the site of inflammation as well as the secretion of additional adhesion factors and cytokines, thereby limiting the infiltration of inflammation. The effects of the IL-1 receptor antagonist, anakinra have been evaluated in severe/critical COVID-19 patients. Overall, anakinra improved clinical conditions, decreased the need for mechanical ventilation and reduced mortality (97–99). As for canakinumab, it reduced systemic inflammation and improved oxygenation in non-ICU patients with mild or severe COVID-19 (100, 101). In critical COVID-19 patients, elevated IL-17 levels have been shown to stimulate neutrophil recruitment and induce inflammatory process and cellular injury. Therefore, IL-17 has been proposed to be a potential therapeutic target for COVID-19. Currently, there are three FDA-approved IL-17 blocking agents, namely secukinumab, ixekizumab and brodalumab used for psoriasis (22, 102–104). A retrospective multicenter observational study found that patients prescribed immunomodulatory drugs for psoriasis, including IL-17A inhibitors, had low hospitalization and no deaths from COVID-19 (105).

Other than that, IL-6 and TNF-α inhibitors have been demonstrated to act on IL-6 and TNF-α immune receptors and thus inhibit the JAK/STAT3 or NF-κβ signaling pathway, which activates the pro-inflammatory genes. This prevents the production of various acute phase proteins such as C-reactive protein (CRP), fibrinogen, thrombopoietin and ferritin, therefore mitigating immune hyperactivation (106). Patients who are already on anti-TNF therapy for other indications demonstrated decreased rate of COVID-19 associated hospitalization and death compared to the other immune-suppressing medications (107, 108).

Initial single-arm studies with IL-6 receptor antagonist, tocilizumab showed a reduction in mortality (109). Treatment with another IL-6 receptor antagonist, sarilumab improved respiratory parameters (110). Despite the initial positive outcomes, early randomized trials with IL-6 receptor antagonists showed mixed results in patients with varying degrees of Covid-19 disease. One randomized trial with tocilizumab significantly reduced the need for ventilation. However, no difference in mortality was observed between the treatment and control groups (111). Another study with tocilizumab showed a significant reduction in the rate of mechanical ventilation or death by day 28, but it did not improve survival (112). In a clinical trial involving critically ill patients, treatment with tocilizumab or sarilumab improved outcomes, including 90-day survival (113). In another trial involving patients with hypoxia and systemic inflammation, tocilizumab reduced mortality and intubation rates (114). It is important to note that the majority of patients in these two trials were receiving steroids, supporting the benefits of combining IL-6 modulation with corticosteroids for COVID-19.

Immunomodulatory therapies can be very beneficial for the treatment of COVID-19 and to improve its clinical severity mediated by the hyperactive immune response. However, the administration of immunomodulatory therapies requires a limited window of opportunity at the onset of hyperinflammation, but before the disease becomes fulminant. In addition, large randomized controlled trials are required to evaluate the potential risks of immunosuppression and adverse effects of immunomodulatory therapies.

## Conclusion

As demonstrated above, the progressive nature of COVID-19 is characterized by its hyperinflammatory effect. The pediatric population has mostly been left unscathed with only mild to moderate disease trajectory (115). Recently, however, a rare but critical complication of COVID-19 known as the multisystem inflammatory disease in children (MIS-C) has been reported in a small subset of children about 2-6 weeks post-SARS-CoV-2 infection (115). Presentations include persistent fever for at least 24 hours and multi-organ impairment involving gastrointestinal, dermatological, neurological, renal, respiratory, cardiac and/or hematological systems (115). In comparison to non-MIS COVID-19 patients, MIS-C displayed more pronounced T cell activation and proliferation, particularly the CD8+ T cells, as well as prolonged and altered plasmablast responses (116). MIS-C also had marked T cell lymphopenia compared to its other COVID-19 pediatric counterpart (116).

Besides that, the high mutative potential of SARS-CoV-2 arouses a cause of concern. Many virus variants, Alpha and Eta from the United Kingdom, Beta from South Africa and Gamma from Brazil have been identified and conferring higher risk than its predecessor, with increased transmissibility and severity (117, 118). Although there may be cross-reactivity between variants, vaccination responses can prove challenging since vaccines are pivotally derived based on the viral spike protein (118). Many, if not all of the SARS-CoV-2 variants contain some form of mutation on its spike protein. For instance, spike protein deletion is seen in the Alpha variant, while the Beta and Gamma present E484K mutation in their spike protein (118).

As more clinical and molecular data accumulates, it is getting progressively clear of the multisystem implications of COVID-19 even beyond hospital discharge. Multidimensional research focusing on the underlying mechanisms of the various SARS-CoV-2 variants and their inflammatory profiles across the disease spectrum is warranted to provide better COVID-19 therapy and prevent vaccination escape.

## Author Contributions

Conceptualization by LT and VR. Methodology by LT. Writing—original draft preparation by LT. Writing, review and editing by LT, TK, and VR. Supervision by VR. All authors contributed to the article and approved the submitted version.

## Conflict of Interest

The authors declare that the research was conducted in the absence of any commercial or financial relationships that could be construed as a potential conflict of interest.

## Publisher’s Note

All claims expressed in this article are solely those of the authors and do not necessarily represent those of their affiliated organizations, or those of the publisher, the editors and the reviewers. Any product that may be evaluated in this article, or claim that may be made by its manufacturer, is not guaranteed or endorsed by the publisher.
